# Assessing heatwave effects on disabled persons in South Korea

**DOI:** 10.1038/s41598-024-54015-x

**Published:** 2024-02-11

**Authors:** Yeji Kang, Ingul Baek, Jongchul Park

**Affiliations:** Kongju National University, 56 Gongjudaehak-Ro, Gongju, 32588 South Korea

**Keywords:** Heat wave, Relative risk, Disabled, Heat-related illnesses, Environmental health, Environmental impact, Risk factors, Natural hazards, Climate-change adaptation

## Abstract

This study investigated the risk of heatwaves for people with disabilities and other socioeconomic attributes using Health Care Bigdata in South Korea. The Health Care Bigdata provides detailed information on heat-related illness (HRI) patients in 2011–2020 from seven major cities. We employed the Distributed Lag Nonlinear Model (DLNM) to measure heat waves' relative risk. Our findings are four-fold. First, the relative risk (RR) of disabled people was 5.075 (95% confidence interval 4.476–5.674), significantly surpassing that of non-disabled people, 3.296 (2.517–4.075). Second, among various personal characteristics studied, disability influenced RR the most, exceeding impacts from elderly (4.457: 3.748–5.166), low-income (3.909: 3.004–4.813), and outdoor (4.052: 2.940–5.164). Third, the disabled young group (5.305: 4.414–6.195) was more vulnerable than the non-disabled elderly group (4.287: 3.576–4.999). Lastly, no significant difference in relative risk was observed between the mild (4.413: 3.855–4.971) and severe disabled groups (4.013: 3.121–4.905).

## Introduction

In recent decades, global warming has exacerbated heat waves' intensity, frequency, and duration, posing significant risks to public health. Besides longer duration, the irregularity in the occurrence of heatwaves deteriorates the adverse effects of heatwaves^1,2^. South Korea has also been exposed to the effect of heatwaves, which attributed to the increase in the daily maximum temperature by 1.5 ℃ and the number of days by 6.9 days observed from 1973 to 2019. South Korea in 2018 experienced record-high temperatures across various climate indices^3^.

Many countries have witnessed a significant increase in mortality by heat waves since 2000. Notably, the devastating heatwave 2003 resulted in 72,210 deaths across 15 European countries^4^. There were 892 and 1,462 excess deaths in the UK and France in 2019, respectively^5^. Furthermore, in 2022, there were 61,672 deaths in 35 European countries, with approximately 18,000 in Italy and 11,300 in Spain^6^. In South Korea, the heatwave of 1994 led to an estimated 3,384 deaths, including 929 excess deaths, in 2018^7,8^.

A strand of literature has explored the health vulnerabilities and associated mortality risks during heat waves. For example, Excessive exposure to heatwaves heightens susceptibility to heat-related illness^9^, cardiovascular diseases^10^, respiratory diseases^11^, mental-related diseases^12^, and infectious diseases^13^. Mora et al*.* argue that there are at least 27 pathways through which heat waves can lead to fatalities^14^. Even the impact of heat waves on health conditions is concentrated on specific socioeconomic cohorts. Older adults^15–17^, Outdoor workers^18–20^, low-income^21,22^, and single-person household^23,24^ are known to be a relatively vulnerable group to heatwaves.

Despite the importance of the investigation on the various cohorts susceptible to heatwaves, to the best of our knowledge, there are mere studies that are interested in the relative risks of disabled people compared to other vulnerable groups. Semenza et al*.* showed that for heat-related death, the odds ratio for people confined to bed was 5.5 compared with those not confined to bed in the Chicago 1995 heat wave^23^. During the same period, the risk of heat-related death for people needing nurse assistance was approximately six times (odds ratio, 6.2) higher than for healthy people. During the 2003 French heat wave, the mortality rate of bedridden patients was 5.5 times higher than that of non-bedridden patients in nursing homes for older adults^25^.

Nevertheless, a battery of studies has provided limited evidence on the relative vulnerability of disabled people to heat waves because of the small size of collected data with limited area and short time horizon. Our study fills this gap by exploiting the dataset established by Health Care Bigdata of South Korea, which provides information on visitors to medical institutions. The dataset provides several interesting characteristics of observables, which enable us to explore the relative risk of exposure to heatwaves by distinct socioeconomic groups classified by disability, region, age, income level, and work environment.

This paper embarks on an empirical investigation into the repercussions of heatwaves, primarily focusing on disabled individuals among other cohorts. Our research strategy entails, firstly, assessing heatwave risks in individuals with and without disabilities. Age-specific group analyses will be conducted to address potential endogeneity problems. Following this, we compare the relative risks of the disabled population and other heat-wave vulnerable groups. Finally, we aim to discern the differential impacts on individuals with varying degrees of disability.

## Materials and methods

### Data source and study population

Our study drew upon a tailored database within the Health Care Bigdata provided by the National Health Insurance Service in South Korea. This data encompasses both outpatients and inpatient admissions diagnosed with heat-related illnesses, as per the International Classification of Diseases, 10th Edition (ICD-10: T67), spanning from 2011 to 2020. The cities covered in our study are Seoul, Busan, Daegu, Incheon, Gwangju, Daejeon, and Ulsan.

Table 1 presents the number of patients affected by heat-related illnesses (HRI). Across the decade, 296,829 individuals were diagnosed with HRI. Among them, 46,393 were aged 65 or older, while 250,436 were under 65. The dataset also included 13,735 disabled patients, segmented into 4,106 with severe disabilities and 9,629 with mild disabilities. The low-income and outdoor worker comprised 51,653 and 33,159 individuals, respectively.

**Table 1:** Number of HRI patients used in the study, 2011–2020.

Group	Elderly	Young	Sum
All	46,393	250,436	296,829
Disabled	6920	6815	13,735
Non-disabled	39,473	243,621	283,094

### Ethical approval

Our study protocol underwent thorough review and received approval from the Institutional Review Board (IRB) of Kongju National University (Confirmation No. 2022-46). Given the observational nature of this study and the utilization of anonymized statistical data, the IRB committee dispensed with the need for informed consent. All methodologies conformed to the guidelines stipulated by the Korean government concerning health and medical data usage.

### Socio-economic classification

Participants were categorized based on socio-economic determinants into elderly, youth, outdoor workers, low-income individuals, and disabled groups. Those aged 65 and above were labeled as elderly, while those below this threshold were termed youth. The outdoor worker category encompasses professionals across sectors, including agriculture, hunting, forestry, fishing, mining, electricity, construction, and manufacturing. The low-income bracket was constituted of individuals in the lowest quintile for insurance premiums and medical aid beneficiaries. The type of disabilities defined officially by National Health Insurance Service includes people with physical disabilities, brain lesions, blind people, other disabilities (language, intellectual, autistic, mental, kidney, heart, respiratory tract, liver, face, ostomy, urinary tract, epilepsy) and national merit with related disabilities. Disability ratings follow the disability rating system and the criteria for determining the degree of disability in Korea. Korea is classified into severe (grades 1–3) and mild (grades 4–6) from 2019.

### Weather data

The weather data were sourced from ASOS (The Automated System Observing System) for each city provided by the Korea Meteorological Administration. Our analyses incorporated daily maximum temperature (T_max_) and daily average humidity.

### Empirical methodology

The statistical analysis was divided into two-stages. In the first stage, time-series regression was applied to each city in order to derive estimates of location-specific associations between heat-related illnesses and ambient temperature, reported as relative risk (RR). DLNM (Distributed Lag Nonlinear Model) was employed to analyze the relationship between heat-related illnesses and ambient temperature. DLNM has been widely used in epidemiological^26–29^. The key features of the DLNM is nonlinearity, time lagged effects, and control for confounders. The model allows capturing nonlinear relationships between the exposure and outcome variables. The model account for delayed effects of exposures on outcomes. The model can incorporate adjustments for potential confounding factors or time-varying covariates. The variable composition of the model for this study is as follows:

$${\text{ln}}{y}_{t}=\alpha +\beta \times Tma{x}_{t-3}+N{S}_{1}\left(s{n}_{t}\right)+N{S}_{2}\left(rhav{g}_{t}\right)+N{S}_{3}\left(do{y}_{t}\right)+\gamma \times weekda{y}_{t}+{\varepsilon }_{t},$$

where ${y}_{t}$ denotes the number of patients with heat-related illnesses, $rhav{g}_{t}$ is the daily average humidity, $do{y}_{t}$ is the day in a year, and $s{n}_{t}$ is the serial number. The degree of freedom of $s{n}_{t}$ is set to 6 times the spanning time of the data, 10-year. $weekda{y}_{t}$ is the day of the week, Natural cubic spline (*NS*) shows a nonlinear relationship between dependent and independent variables. Other than the variables at time $t$, a daily maximum temperature, $Tma{x}_{t-3}$, is the 3-day lagged times series. The use of the time-lagged series is in line with Hess et al*.*, Heo et al*.*, and Royé et al*.*, which provide evidence that the impact of heat waves strongly remains on the period^26–28^.

Once the DLNM is fitted, the estimated coefficients obtained from the model can be used to calculate the relative risk. The DLNM estimates a log-linear relationship between exposure and outcome, the relative risk can be derived by exponentiation the coefficient associated with the exposure variable, as follow.

$$RR={e}^{\beta }$$

β is the estimated coefficient from the DLNM representing the log-relative risk. The relative risk measures the ratio of patient occurrence over certain temperature while holding all other confounding factors.

To compare relative risks between groups or cities, we converted nonlinear RRs to the average RR above the 90th percentile daily maximum temperature. The daily maximum temperature at which the number of patients begins to increase, in other word, the threshold temperature, varies for each city depending on location-specific temperature range. We found that the threshold temperature was similar to approximately the 90th percentile daily maximum temperature for each city (You can see this in the results). Therefore, to express the nonlinear RR as a quantitative effect size to facilitate comparison between cities or groups, and to use it as an effect size for heat waves in the next stage, we averaged the RR above the 90th percentile daily maximum temperature.

In the second stage, meta-analysis was applied to synthesize and analyze information across multiple cities. In meta-analysis, we pooled the RRs from the first stage to calculate a more accurate or comprehensive relative risk. The weight assigned to each effect size (means the RR above the 90th percentile Tmax in this study) from location-specific time-series regression analysis reflects the contribution to the overall effect estimate. The weight for an effect size was determined by the inverse variance method in this study. In the meta-analysis, a fixed-effect model and a random-effect model are employed to estimate the average effects. The fixed-effect model is for data with characteristics of homogeneity on population while the random-effect model is for heterogeneity. Q statistics and Higgins' I2 are applied for the homogeneity test for the population. The homogeneity tests were conducted using Q statistics and Higgins's I^2^. Due to the limited number of cases in the meta-analysis, homogeneity was considered significant if the p-value in the Q-test was less than 0.1 and if I^2^ exceeded 50% (Table 2).

**Table 2:** The test result for homogeneity within each separate group.

Group	p-value	Higgins's I^2^ (%)	Meta-analysis model
Disabled			
All	0.30	17	Fixed-effect model
Elderly	0.33	13	
Young	0.66	0	
Severe	0.27	21	
Mild	0.16	35	
Non-disabled			
All	0.01	98	Random-effect model
Elderly	0.01	80	
Young	0.01	98	
Vulnerable groups			
Elderly	0.01	78	
Low-income	0.01	89	
Outdoor	0.01	93	

The first-stage regression was performed with the R software (version 4.1.2), using functions in the package *dlnm* (version 4.3.0). The package *meta* (version 6.5.0) of the R was used for the second-stage meta-analysis.

## Results

This chapter presents our primary findings, which are derived from a comparative analysis of relative risks across cities and a meta-analysis based on the response curve of relative risk and daily maximum temperature. To ensure compatibility and representativeness, we average the relative risks for temperatures exceeding the 90th percentile, as risks tend to increase beyond this threshold. This chapter analyzes the relative risks of disabled individuals against several groups: the non-disabled, older adults, low-income individuals, and outdoor workers. Furthermore, we delve into the differences in relative risks based on the severity of disability.

### Comparison of disabled and non-disabled groups

**Figure 1:**
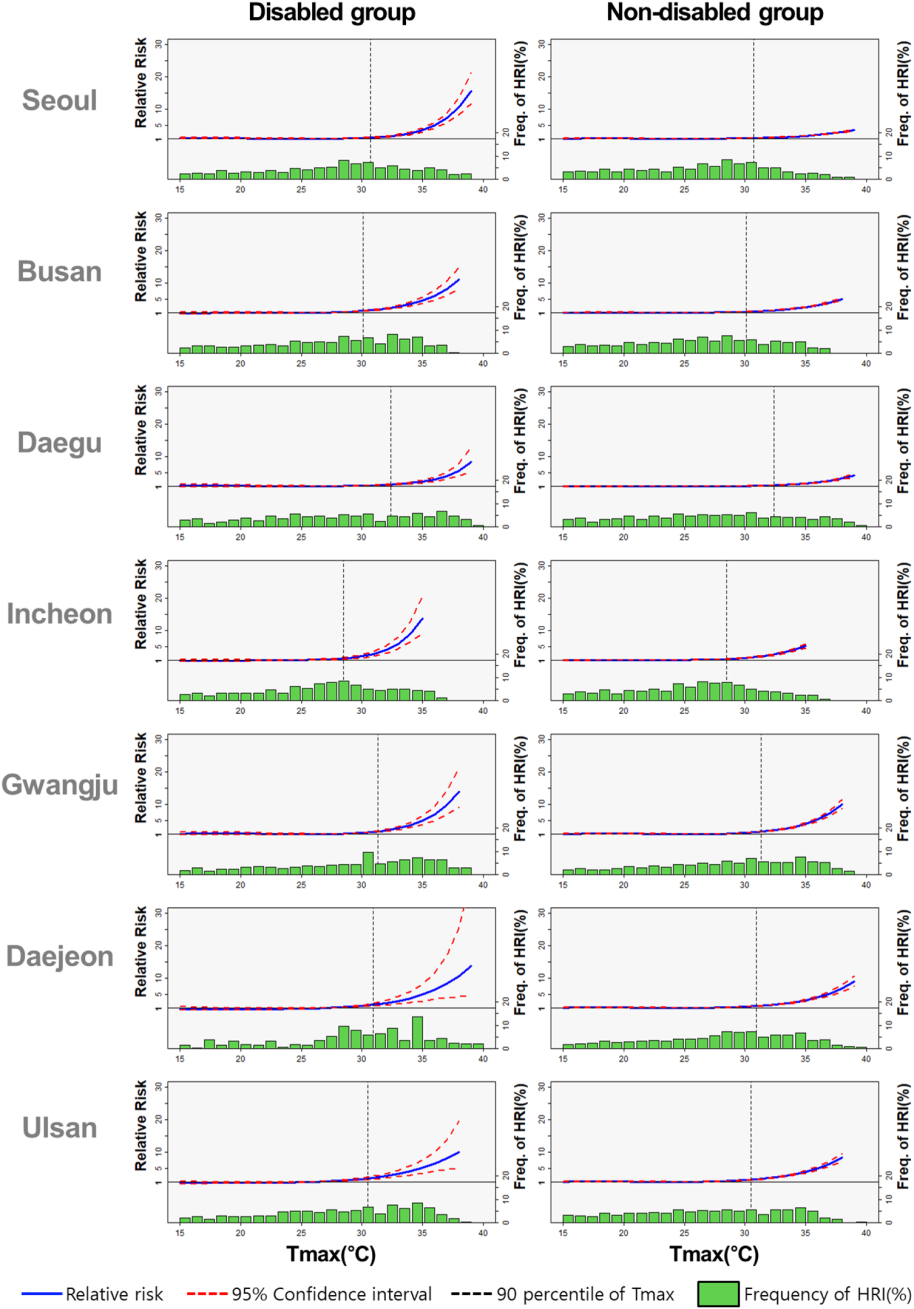
Comparison of the relative risk between disabled and non-disabled groups.

The relative risks of HRI patients begin to increase beyond the 90th percentile of temperature across disabled and non-disabled groups in common. However, Fig. 1 displays that both groups have significant differences in response to the change in temperature beyond the threshold. The relative risk, measured based on the number of HRI patients, exponentially increased as the temperature rose in the disabled group.

The relative risk of HRI for the disabled group was 1.6 times higher than its counterpart on average. Table 3 shows the average relative risk beyond the threshold of both groups across cities. Specifically, the relative risk of HRI patients with disability and non-disability in Seoul was 5.532 (4.409–6.967) and 2.076 (1.976–2.181), respectively. Across all other cities, disabled groups are exposed to higher risks in heat waves in terms of relative risk. Following the result of the meta-analysis, heatwaves severely affected the disabled group. The differences in both groups in response to heatwaves are statistically significant because the confidence intervals do not overlap.

**Table 3:** Average RR above the 90th percentile T_max_ for each city in the disabled and non-disabled groups.

City	Disabled group			Non-disabled group		
	All	Young	Elderly	All	Young	Elderly
Seoul	5.532 (4.409–6.967)	5.526 (3.871–7.969)	5.223 (3.971–6.905)	2.076 (1.976–2.181)	1.879 (1.780–1.985)	3.114 (2.766–3.511)
Busan	5.047 (4.029–6.348)	6.420 (4.684–8.863)	3.979 (2.884–5.538)	2.766 (2.603–2.940)	2.554 (2.390–2.73)	4.110 (3.598–4.703)
Daegu	3.874 (2.886–5.252)	4.320 (2.844–6.684)	3.509 (2.320–5.416)	2.414 (2.215–2.634)	2.222 (2.022–2.444)	3.694 (3.019–4.539)
Incheon	5.625 (4.035–7.866)	4.399 (2.968–6.596)	6.534 (4.252–10.15)	2.814 (2.580–3.071)	2.616 (2.381–2.876)	4.281 (3.463–5.310)
Gwangju	6.318 (4.580–8.785)	6.492 (4.137–10.336)	6.494 (3.934–10.976)	4.892 (4.421–5.417)	4.601 (4.118–5.145)	6.184 (4.877–7.874)
Daejeon	7.269 (4.322–12.693)	6.155 (3.076–13.216)	6.508 (3.515–12.518)	4.080 (3.599–4.633)	3.912 (3.409–4.498)	4.968 (3.674–6.781)
Ulsan	5.351 (3.454–8.472)	6.340 (3.726–11.038)	4.480 (2.445–8.554)	4.184 (3.77–4.647)	4.050 (3.620–4.535)	5.026 (3.802–6.689)
Meta-analysis	5.075 (4.476–5.674)	5.305 (4.414–6.195)	4.566 (3.821–5.311)	3.296 (2.517–4.075)	3.095 (2.322–3.867)	4.287 (3.576–4.999)

**Figure 2:**
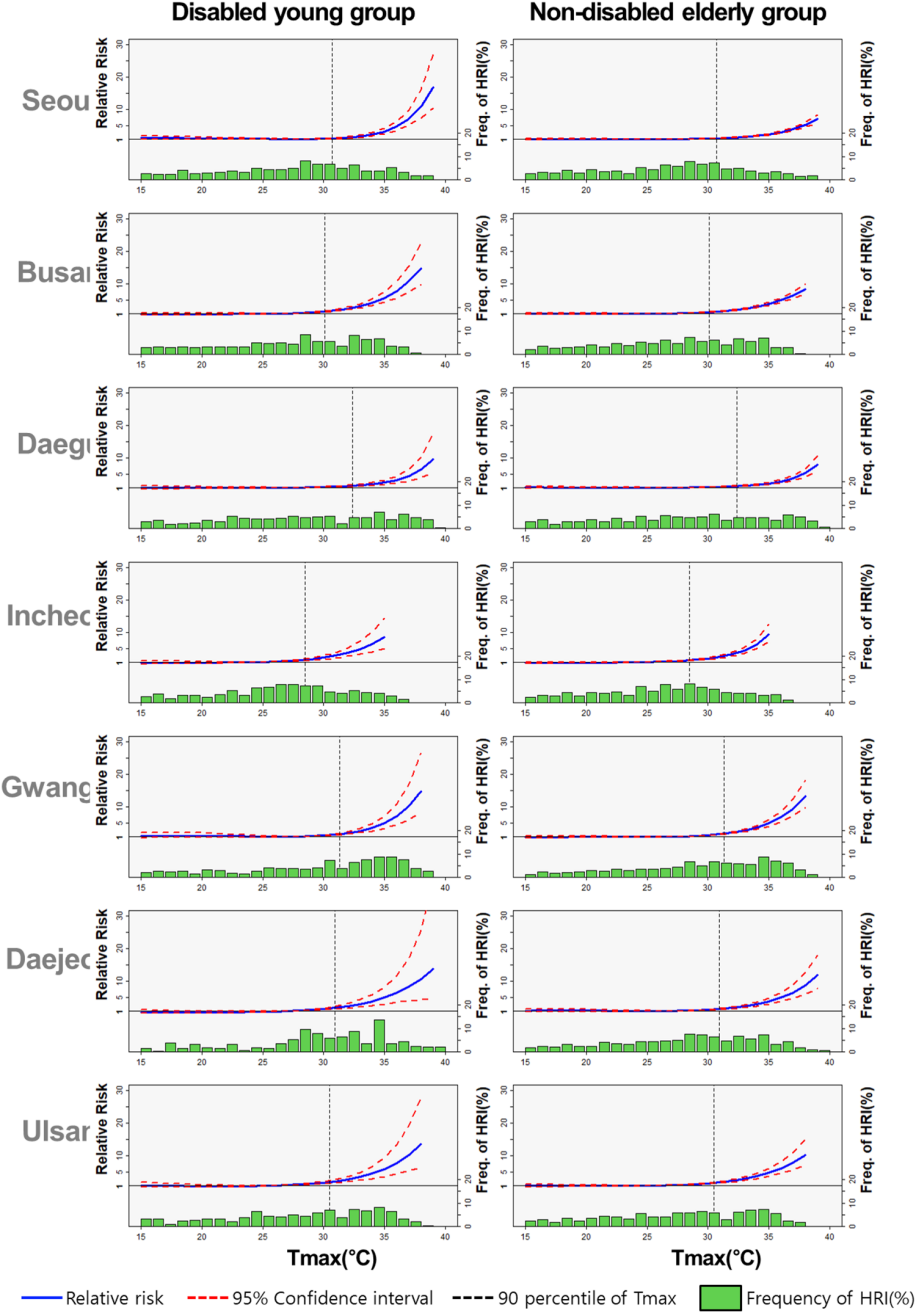
Comparison of the relative risk in the disabled young group and the elderly group without disabilities.

Table 3 also shows two fruitful findings. First, disability is a significant factor that increases the risk of heat waves along with age. Remarkably, the relative risk of the disabled young group was higher than that of the non-disabled elderly group across all cities, measured at 5.305 (4.414–6.195) and 4.287 (3.576–4.999), respectively, in the meta-analysis. Figure 2 presents the general tendency and relative risks in Seoul, Busan, and Ulsan accelerated over the daily maximum temperature horizon. Second, the aging effect of heatwave can be differentiated by disability based on the meta-analysis. On the contrary, older adults tend to be more vulnerable to the risk than young people in the non-disabled group (4.287: 3.576–4.999 and 3.095: 2.322–3.867, respectively). While vulnerability by age in non-disabled individuals is a well-documented fact in existing studies, the notably high vulnerability among young people with disabilities is an intriguing outcome. This highlights the substantial impact of disabilities on heatwave risk, suggesting that they play a significant role, potentially interacting with age factors.

### Comparison of relative risks among vulnerable groups

The next task in this section is to compare the extent to which social class is the most vulnerable based on the relative risks against heat waves. To do so, we classify the sample by age (older adults), income (low-income), and in-or-outdoor workers as a disabled group, which are highly susceptible to high temperature-related illnesses.

Table 4 points out that, in terms of the measure of the relative risk by the meta-analysis, the disabled group was the most vulnerable group to exposure to scorching across areas. For instance, the relative risk in Seoul is 5.532 (4.409–6.967) for the disabled group, 3.405 (3.050–3.805) for the older adults, 2.834 (2.519–3.194) for the low-income class, and 1.941 (1.695–2.231) for the outdoor workers. From the perspective of regions, the degree of risk in Daejeon is relatively higher than in other cities over characteristics. Across the seven major cities in South Korea, the RR for the disabled is 5.075 (4.476–5.674), 4.457 (3.748–5.166) for elderly, 3.909 (3.004–4.813) for low-income, 4.052 (2.940–5.164) for outdoor workers. Regarding 95% significance level, the RR of the disabled is clearly higher than elderly and low-income, while that of outdoor worker is insignificantly lower. As a result, disabilities are highly significant factor that increases the susceptibility of heatwaves.

**Table 4:** Relative risk and meta-analysis results by socio-economically vulnerable group.

City	Disabled	Elderly	Low-income	Outdoor
Seoul	5.532 (4.409–6.967)	3.405 (3.050–3.805)	2.834 (2.519–3.194)	1.941 (1.695–2.231)
Busan	5.047 (4.029–6.348)	4.126 (3.645–4.676)	3.985 (3.527–4.508)	4.437 (3.689–5.347)
Daegu	3.874 (2.886–5.252)	3.660 (3.052–4.405)	2.674 (2.289–3.129)	3.138 (2.566–3.857)
Incheon	5.625 (4.035–7.866)	4.694 (3.873–5.703)	3.029 (2.495–3.684)	3.321 (2.650–4.172)
Gwangju	6.318 (4.580–8.785)	6.189 (4.997–7.693)	5.348 (4.436–6.465)	6.016 (4.646–7.829)
Daejeon	7.269 (4.322–12.693)	5.254 (4.004–6.944)	6.119 (4.689–8.038)	5.924 (4.043–8.811)
Ulsan	5.351 (3.454–8.472)	5.110 (3.845–6.854)	4.387 (3.423–5.653)	5.006 (3.870–6.496)
Meta-analysis	5.075 (4.476–5.674)	4.457 (3.748–5.166)	3.909 (3.004–4.813)	4.052 (2.940–5.164)

### Comparison of relative risks between severe and mild disabled groups

**Figure 3:**
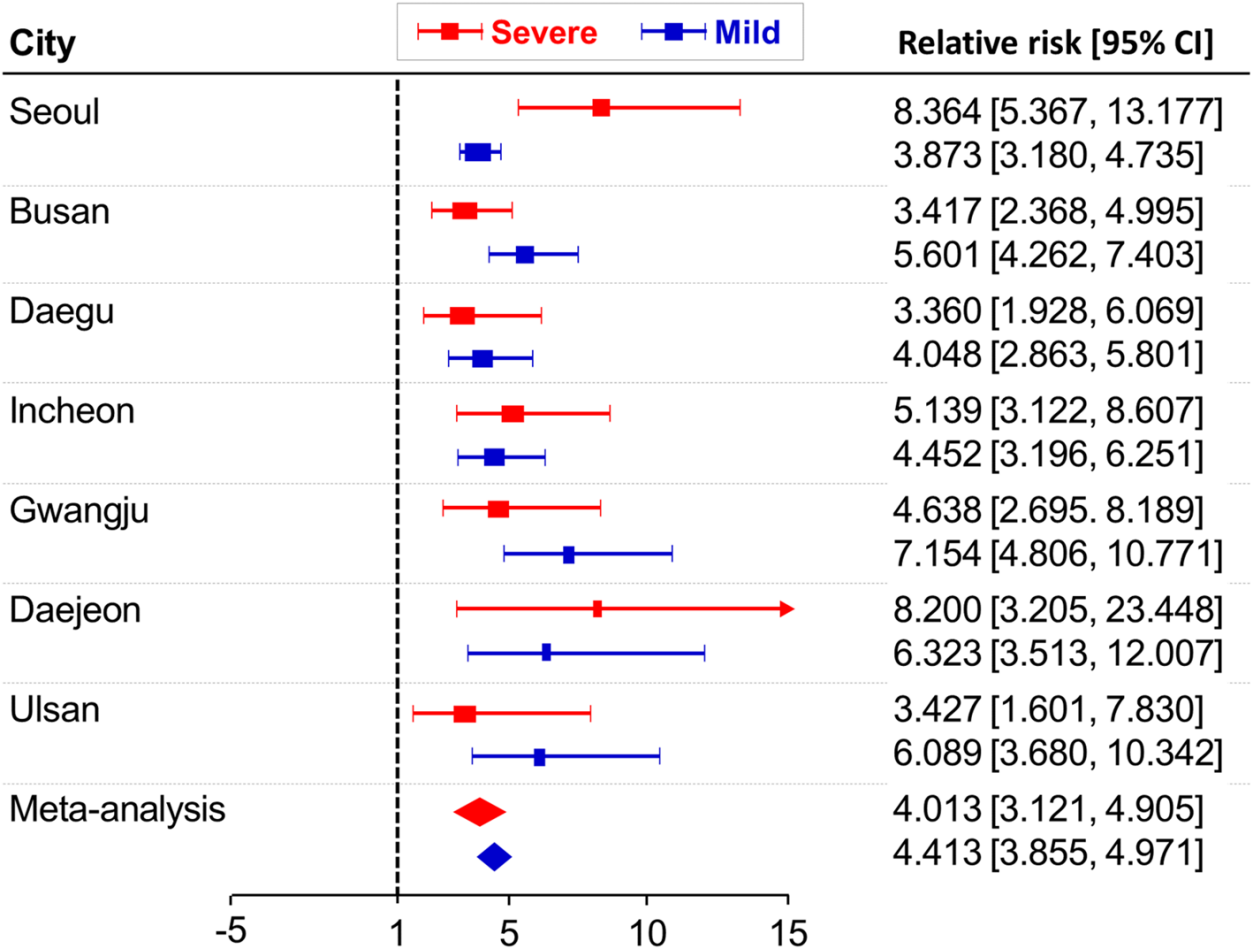
Relative risk and meta-analysis results according to severity of disability.

We partitioned the disabled group in the sample by the degree of the disability into two groups: the mild-disabled group and the severe-disabled group. Figure 3 demonstrates regional heterogeneity depending on the severity of disability in terms of the relative risk. The group with severe disabilities had a higher relative risk than those with mild disabilities in Seoul, Incheon, and Daejeon. On the contrary, in Busan, Daegu, Gwangju, and Ulsan, people with mild disabilities had more damage during heat waves.

Specifically, in Seoul, the relative risk of a group with severe and mild disabilities was 8.364 and 3.873, respectively, but in Ulsan, those numbers were 3.427 and 6.089, respectively. The meta-analysis result shows that RRs in the mild and severe disability groups are 4.413 (3.855–4.971) and 4.013 (3.121–4.905), respectively. Therefore, no distinct difference is found in RRs in the two groups.

## Conclusions and discussion

In this study, we confirmed the quantitative significance by measuring the vulnerability to heatwaves by measuring the relative risk of not only disabled people but also the other groups with distinct socioeconomic characteristics using Health Care BigData. Our empirically estimated results bolster the previous studies that the relative risk of the disabled group was found to be about higher than that of the non-disabled group. We also found that, by segregating the sample into socioeconomic criteria, people with disabilities were the more vulnerable group rather than groups of older adults, low-income class, and outdoor workers. This result suggests that special attention to disabled people is needed in social policies against heatwaves.

Previous studies on the socio-economic characteristics of people with disabilities have provided clues to understand their vulnerability to extreme heat. Firstly, research on the relative poverty of people with disabilities has been conducted. According to a study that elucidated the process by which individuals with disabilities migrated into impoverished slum villages with inadequate living conditions, disability reduces the labor force, making it difficult to enter the labor market. And additional costs due to disability intensify economic difficulties. It is difficult for disabled people to escape poverty, and they eventually fall into a state of chronic poverty^30^. In Korea, the poverty rate among people with disabilities is three to four times higher than that of non-disabled individuals^31^. The economically disadvantaged face significant constraints in energy usage to avoid extreme heat due to financial reasons^32^.

Second, several studies have highlighted that disability reduces relative accessibility to health care. The disabled group faced constraints in accessing medical services due to barriers in physical accessibility, a lack of coordinated care, and insufficient training for healthcare professionals as well as communication barriers^33,34^.

Third, people with disabilities may also face difficulties in accessing risk information. Depending on the type of disability, individuals may have diverse approaches to accessing information. However, due to the lack of consideration for the diversity in information dissemination by disaster planners, individuals with disabilities might find it challenging to acquire heat-related information^35^. Groups receiving heat-related information statistically exhibit significant differences in heat avoidance behavior compared to those who do not receive such information^36^. Therefore, if heat-related information is not effectively communicated to individuals with disabilities, there might be limitations in inducing heat avoidance behaviors among them.

Lastly, people with disabilities can be marginalized from public services provided through heatwave policies. A representative heatwave adaptation policy in Korea is the cooling center. Research findings in Korea revealed that individuals facing both physical disabilities and poverty encountered difficulties accessing nearby cooling center^37^.

Summarizing existing research, individuals with disabilities are more likely to face relative poverty and inadequate living conditions. Limited accessibility to healthcare services can pose challenges in managing their health, potentially increasing the risk of health issues due to exposure to heat during heatwaves. Additionally, the low accessibility to public services that could mitigate the impact of heat, such as warning systems and cooling centers, exacerbates the heat-related risks for people with disabilities.

The main finding is that people with disabilities, especially younger individuals with disabilities, are exposed to the risk of high temperatures. This result suggests that the socio-economic factors mentioned above, which contribute to heat vulnerability among individuals with disabilities, apply to both younger and older disabled individuals. Furthermore, it indicates a need for improvement in current heatwave policies concerning individuals with disabilities.

The comparison between mild- and severe-disabled groups suggests no significant difference. However, according to Holstein et al., the risk of people with mild disabilities was higher than that of people with severe disabilities^25^. They found that, during the 2003 European heat wave, mortality rates increased significantly: low-dependence patients saw an 8.3-fold rise, while high-dependence patients experienced a 3.9-fold increase in French nursing homes for older adults. The difference between the two results may be due to differences in the severity of health effects. One is for deaths and the other is for outpatients and hospitalizations. To substantiate the concept of heatwave effects differing by disability severity, more compelling evidence from future research is needed. Continuing research might help clarify these differences and provide a more comprehensive understanding of heatwave impacts on various disability levels.

Our study has four limitations on the analysis: subgroup analysis, consideration of disability types, age group classification, and scope of health impact. First, for subgroup comparison, it shrinks the size of samples in a group which would lead to noncredible statistical inference. For instance, in the elderly group, there are various personal characteristics such as low-income, outdoor worker, and people with disabilities. We employ mutually exclusive grouping, subgroup analysis, and group readjustment to solve the issue, but it reduces considerably the size of samples of our interest. Second, when dealing with the vulnerability of the disabled, the type of disability was not sufficiently considered. Third, we do not classify the age-group in detail, which makes it difficult to the exposure risk from the perspective of age distribution. Different age groups may be exposed to heterogeneous risks during heatwaves. Although in our study we divide two age groups based on age 65, it is needed further study on the susceptibility of young people with disabilities by separating age groups in detail on heatwaves. For future studies, in addition to measuring the risk, the cognition and behavior patterns against heatwaves should be studied together. Fourthly, this study focused on outpatient and hospitalized patients with heat-related illnesses, which represent relatively mild consequences of heatwaves. More severe consequences of heatwaves include fatalities or emergency hospitalizations. Continuous research focusing on the types of damages caused by more severe heatwaves is necessary to comprehend the diverse health impacts of heat on individuals with disabilities. Addressing the limitations of such studies will be an ongoing effort to supplement and enhance future research.

This study's findings hold significance in reporting scientific evidence regarding the heatwave risks faced by individuals with disabilities. While this study encountered limitations in explaining the health impacts of heatwaves based on the severity and types of disabilities, it heightened that disabled individuals face higher relative risks than non-disabled and other heatwave-vulnerable populations. Furthermore, our findings emphasize the substantial influence of disability as a key factor in exacerbating heatwave risks; however, it's important to acknowledge that interactions with age factors may contribute to the overall complexity of this relationship. This underscores the necessity for policymakers to include younger individuals with disabilities in the formulation of heatwave policies, ensuring their needs are not overlooked. Such insights hold considerable potential for informing future policy considerations related to heatwave precautions for people with disabilities. The findings of this study are anticipated to provide foundational information to reduce heat risks among individuals with disabilities and establish successful heat response policies specifically tailored for them.

## Data Availability

The data that support the findings of this study are available from Korea's National Health Insurance Service but restrictions apply to the availability of these data, which were used under license for the current study, and so are not publicly available. Final analysis data however could be available from the authors upon reasonable request and with permission of the National Health Insurance Service. Please contact the corresponding author (Jongchul Park, jcp@kongju.ac.kr), if someone wants to request the data from this study and submit the amendment manuscript.
